# Persistence of salivary antibody responses after COVID-19 vaccination is associated with oral microbiome variation in both healthy and people living with HIV

**DOI:** 10.3389/fimmu.2022.1079995

**Published:** 2023-01-10

**Authors:** Mahin Ghorbani, Khaled Al-Manei, Sabrina Naud, Katie Healy, Giorgio Gabarrini, Michal Jacek Sobkowiak, Puran Chen, Shilpa Ray, Mira Akber, Sandra Muschiol, Gordana Bogdanovic, Peter Bergman, Per Ljungman, Marcus Buggert, Hans-Gustaf Ljunggren, Elisa Pin, Piotr Nowak, Soo Aleman, Margaret Sällberg Chen

**Affiliations:** ^1^ Department of Dental Medicine, Karolinska Institutet, Huddinge, Sweden; ^2^ Department of Restorative Dental Science, College of Dentistry, King Saud University, Riyadh, Saudi Arabia; ^3^ Department of Laboratory Medicine, Karolinska Institutet, Huddinge, Sweden; ^4^ Department of Medicine, Huddinge, Karolinska Institutet, Huddinge, Sweden; ^5^ Department of Clinical Microbiology, Karolinska University Hospital, Stockholm, Sweden; ^6^ Department of Clinical Immunology and Transfusion Medicine, Karolinska University Hospital, Huddinge, Sweden; ^7^ Department of Cellular Therapy and Allogeneic Stem Cell Transplantation, Karolinska Comprehensive Cancer Center, Karolinska University Hospital, Huddinge, Sweden; ^8^ Department of Protein Science, SciLifeLab, KTH Royal Institute of Technology, Stockholm, Sweden; ^9^ Department of Infectious Diseases, Karolinska University Hospital, Huddinge, Sweden

**Keywords:** Oral microbiome, mRNA vaccination, saliva, SARS-CoV-2, campylobacter, leptotrichia, gemella, granulicatella

## Abstract

Coevolution of microbiome and immunity at mucosal sites is essential for our health. Whether the oral microbiome, the second largest community after the gut, contributes to the immunogenicity of COVID-19 vaccines is not known. We investigated the baseline oral microbiome in individuals in the COVAXID clinical trial receiving the BNT162b2 mRNA vaccine. Participants (n=115) included healthy controls (HC; n=57) and people living with HIV (PLHIV; n=58) who met the study selection criteria. Vaccine-induced Spike antibodies in saliva and serum from 0 to 6 months were assessed and comparative analyses were performed against the individual salivary 16S ASV microbiome diversity. High- versus low vaccine responders were assessed on general, immunological, and oral microbiome features. Our analyses identified oral microbiome features enriched in high- *vs*. low-responders among healthy and PLHIV participants. In low-responders, an enrichment of Gram-negative, anaerobic species with proteolytic activity were found including *Campylobacter, Butyrivibrio, Selenomonas, Lachnoanaerobaculum, Leptotrichia, Megasphaera, Prevotella* and *Stomatobaculum*. In high-responders, enriched species were mainly Gram-positive and saccharolytic facultative anaerobes: *Abiotrophia, Corynebacterium, Gemella, Granulicatella, Rothia*, and *Haemophilus*. Combining identified microbial features in a classifier using the area under the receiver operating characteristic curve (ROC AUC) yielded scores of 0.879 (healthy controls) to 0.82 (PLHIV), supporting the oral microbiome contribution in the long-term vaccination outcome. The present study is the first to suggest that the oral microbiome has an impact on the durability of mucosal immunity after Covid-19 vaccination. Microbiome-targeted interventions to enhance long-term duration of mucosal vaccine immunity may be exploited.

## Introduction

SARS-CoV-2 infection and replication take place in the oral cavity and saliva (1). The presence of local immune control is important in limiting the viral infection and transmission. A durable mucosal immunity at this site is therefore a highly desired outcome in a COVID-vaccination. One of the most immunogenic COVID-19 vaccines - the BNT162b2 (Comirnaty^®^) mRNA vaccine induces high titres of systemic SARS-CoV-2 spike-specific antibodies (2, 3). In the oral cavity and saliva, it conveys virus-binding antibodies highly correlative to neutralizing capacity. The magnitude of this acquired local immunity however varies among both healthy and immunocompromised vaccinees (4), and the durability of those translocated specific antibodies at the oral mucosa is presently unknown. Recent developments in techniques for microbiome sequencing have enabled a comprehensive analysis of commensal microbiota, with over 700 bacterial species detected in the oral cavity (5). The variants of microorganisms form unique communities, and the oral microbiome composition is believed to be resilient and remain rather stable within individuals longitudinally (6). The normal oral microbiota is continuously in contact with the oral mucosa and plays a role in modulating immune surveillance mechanisms (7). Dysbiotic oral microbiota on the other hand is associated with oral diseases, medical conditions, dietary habits, and lifestyles (8–10). Recently, a negative link between vaccine efficacy and gut microbiota variation linked to BMI and systemic inflammation was reported (11), other studies have reported that decreased seroconversion in BNT162b2 mRNA recipients is associated with antibiotic use (12). Although the gut microbiota is recognized as a key factor in supporting gut homeostasis and health (13), the contribution of oral microbiota to COVID vaccination is still unknown.

In the present study, we hypothesize that oral microbiota composition has a role in the maintenance of the salivary immunity induced by mRNA vaccination. We investigated the salivary microbiota using 16S rRNA sequencing analysis in samples obtained from healthy participants (HC) and people living with HIV (PLHIV). The aim was to characterize salivary microbiota signatures associated with a durable vaccine response capable of persisting at the oral mucosal site.

## Results

### Study design and participants

Eligible participants were healthy controls (HC; n=57) and people living with HIV (PLHIV; n=58) who had received two vaccine doses and fulfilled the study inclusion criteria including baseline and 6-month screenings for negativity for SARS-CoV-2 exposures. As described earlier, all participants seroconverted during the one-month follow-up (day 35) i.e.,14 days after dose 2 in both groups (4). Vaccine-induced spike IgG in serum and saliva among the participants correlated significantly during the 6-month follow-up, although a proportion showed a larger reduction in salivary spike IgG in than others (**Figure S1**). Therefore, we sub-grouped the participants as High- or Low-responders, respectively, based on the expected convalescence spike IgG level at this time-point (14). The subgroup characteristics and vaccine response data are summarized in **Tables 1**, **2**. As shown, Low-responders in both the HC and the PLHIV cohorts demonstrated a reduced ability to maintain salivary IgG responses to the full-length trimeric spike (S.f) as well as the S1 spike antigen during this 6-month follow-up. In PLHIV Low-responders, reduced magnitude and duration of spike-IgG was also found in both saliva and serum through the entire follow-up period (**Table 2**). However, High- and Low responders showed no significant differences in any general or medical variable such as age, gender, diet, BMI, ethnicity, anti-inflammatory medications, or clinical immunological variables (**Table 1**). The peak and long-term spike IgG levels in saliva were both about 6-fold lower in Low-responders (n=29) than in the High-responders (n=28). Similarly, in PLHIV Low-responders (n=38), a significant reduction of anti-spike IgG level both in serum and saliva was noted compared to High-responders (n=20). A detailed assessment on the anti-capsid serology and PCR-reports further confirmed that no break-through infection had occurred during the follow-up that could have influenced these results (**Table 2**).

**Table 1:** Demographics and medical report of study participants.

Variables	HC (n = 57)		P-value	PLHIV (n = 58)		P-value
	High responder(n = 28)	Low responder(n = 29)		High responder(n = 20)	Low responder(n = 38)	
Demographic variables						
**Age ^A^ (years)**	47 (32.3)	58 (30)	0.247	54 (22.3)	54 (18.3)	0.518
**Gender**			0.881			0.258
**Male ^B^**	12 (42.9)	13 (44.8)		9 (45)	23 (60.5)	
**Female ^B^**	16 (57.1)	16 (55.2)		11 (55)	15 (39.5)	
**Age-group**			0.190			0.327
**≥ 60 ^B^ (years)**	7 (25)	12 (41.4)		8 (40)	14 (36.8)	
**40-59 ^B^ (years)**	8 (28.6)	10 (34.5)		8 (40)	21 (55.3)	
**18-39 ^B^ (years)**	13 (46.4)	7 (24.1)		4 (20)	3 (7.9)	
**BMI ^A^ (kg/m^2^)**	25 (7)	25.5 (5)	0.600	25.1 (4.3)	25.2 (5.7)	0.390
**Race**			1			0.721
**Caucasian ^B^**	28 (100)	29 (100)		9 (45)	23 (60.5)	
**Black/African American ^B^**	0 (0)	0 (0)		5 (25)	7 (18.4)	
**Latin ^B^**	0 (0)	0 (0)		5 (25)	7 (18.4)	
**Unknown ^B^**	0 (0)	0 (0)		1 (5)	1 (2.7)	
**Diet**			1			1
**Normal ^B^**	28 (100)	29 (100)		20 (100)	38 (100)	
**Vegetarian ^B^**	0 (0)	0 (0)		0 (0)	0 (0)	
Medical variables						
**Non-steroidal anti-inflammatory drugs**			1			1
**Yes ^B^**	0 (0)	0 (0)		0 (0)	0 (0)	
**No ^B^**	28 (100)	29 (100)		20 (100)	38 (100)	
**IgG baseline ^A^(g/L)**	11 (3.3)	11 (2)	0.293	13.5 (3)	12.5 (5)	0.310
**Lymphocytes baseline ^A^ (x10^9^/L)**	1.8 (1)	1.7 (1)	0.712	1.8 (1.1)	1.6 (2.8)	0.969
**Creatinine baseline ^A^ (µmol/L)**	69 (2.7)	71 (22.5)	0.527	77 (35.5)	83 (32.5)	0.423

Mann–Whitney U test was applied to compare the continuous variables and X^2^ test for analyzing categorical variables using SPSS version 24.0 (IBM Crop, Armonk, NY, USA) software. **^A^** variable is illustrated as the median (IQR), whereas **^B^** variable is illustrated as the number (%). IQR, interquartile range.

**Table 2:** Virological data, serum and saliva antibody status of study participants.

Variables	HC (n = 57)		P-value	PLHIV (n = 58)		P-value
	High responder(n = 28)	Low responder(n = 29)		High responder(n = 20)	Low responder(n = 38)	
Virological data						
**PCR-positive at baseline**			1			1
Yes ^B^	0 (0)	0 (0)		0 (0)	0 (0)	
No ^B^	28 (100)	29 (100)		20 (100)	38 (100)	
**Antibody-positive at baseline**			1			1
Yes ^B^	0 (0)	0 (0)		0 (0)	0 (0)	
No ^B^	28 (100)	29 (100)		20 (100)	38 (100)	
**CD4 count**	NA	NA	NA			0.765
≤ 300 ^B^ (cells/mm^3^)	NA	NA		6 (30)	10 (26.3)	
> 300 ^B^ (cells/mm^3^)	NA	NA		14 (70)	28 (73.7)	
Serum antibody level (AU)						
**Anti-S d0 ^A^**	0.4 (0)	0.4 (0)	1	0.4 (0)	0.4 (0)	1
**Anti-S d10 ^A^**	0.8 (1.4)	0.4 (0.9)	0.090	0.4 (1.6)	0.9 (1.7)	0.395
**Anti-S d21 ^A^**	57.4 (119.9)	62.4 (149)	0.786	43.4 (81.6)	19.6 (39.7)	0.015*
**Anti-S d35 ^A^**	2368.5 (1189)	1625 (2878.5)	0.231	1972 (1526)	1049 (1338)	0.005*
**Anti-S mo.6 ^A^**	739.5 (691.3)	559 (737.5)	0.078	782 (967)	249 (412.5)	<0.001*
**Positive seroconversion**			0.999			1
**d.35** Yes ^B^	28 (100)	29 (100)		20 (100)	38 (100)	
**mo 6.**Yes ^B^	28 (100)	28 (96.6)		20 (100)	38(100)	
Saliva antibody level (MFI)						
**Anti-S-f d.0** **Over cutoff (55 MFI)** ^B^	0 (0)	0 (0)	1	0 (0)	0 (0)	1
**Anti-S-f d.10 ^A^ (MFI)**	78 (306)	30 (32)	0.003*	78.5 (34.8)	41 (65.3)	0.098
**Anti-S-f d.21 ^A^ (MFI)**	540 (654)	203 (415)	0.028*	340 (438.8)	159 (315.5)	0.012*
**Anti-S-f d.35 ^A^ (MFI)**	3248 (3287)	571 (1994.5)	<0.001*	3875 (6557)	1251 (2166.5)	0.001*
**Anti-S-f mo.6 ^A^ (MFI)**	438.00 (402.50)	68. (86.5)	<0.001*	567 (362.5)	116.5 (120)	<0.001*
**Anti-S1 d.0** **Over cutoff (98 MFI) ^B^**	0 (0)	0 (0)	1	0 (0)	0 (0)	1
**Anti-S1 d.10 ^A^ (MFI)**	52 (34)	32 (18)	0.003*	53 (66)	38 (49.3)	0.105
**Anti-S1 d.21 ^A^ (MFI)**	332 (426)	161 (273)	0.076	244.5 (308)	107 (163.5)	0.020*
**Anti-S1 d.35 ^A^ (MFI)**	2512 (2141)	400 (1618)	0.001*	2362 (4715.8)	941 (1859.5)	0.004*
**Anti-S1 mo.6 ^A^ (MFI)**	273 (216)	56 (59.5)	<0.001*	321.5 (200)	85 (61.3)	<0.001*
Fold change relative to baseline						
**Anti-S-f d.10-fold change ^A^**	2.3 (10.6)	1.1 (0.9)	0.010*	2.3 (4.6)	1.3 (2.2)	0.351
**Anti-S-f d.21-fold change ^A^**	18 (22.5)	7.8 (13.4)	0.028*	12.4 (13.7)	5.4 (11.4)	0.033*
**Anti-S-f d.35-fold change ^A^**	102 (116.8)	23.8 (67.3)	0.002*	133.5 (274.8)	37.2 (66.5)	0.002*
**Anti-S-f mo.6-fold change ^A^**	15.2 (15.4)	2.5 (3)	<0.001*	18 (15.4)	4.2 (4)	<0.001*

Mann–Whitney U test was applied to compare the continuous variables and X^2^ test for analyzing categorical variables using SPSS version 24.0 (IBM Crop, Armonk, NY, USA) software. **^A^** variable is illustrated as the median (IQR), whereas **^B^** variable is illustrated as the number (%). * Bolded values denotes statistical significance when P-value is < 0.05. NA, not applicable; IQR, interquartile range; d, day; mo, months; S1, spike antigen; S-f, full-length trimeric spike; MFI, mass fluorescence intensity.

### Microbiome richness and diversity in saliva of participants

**Figure 1:**
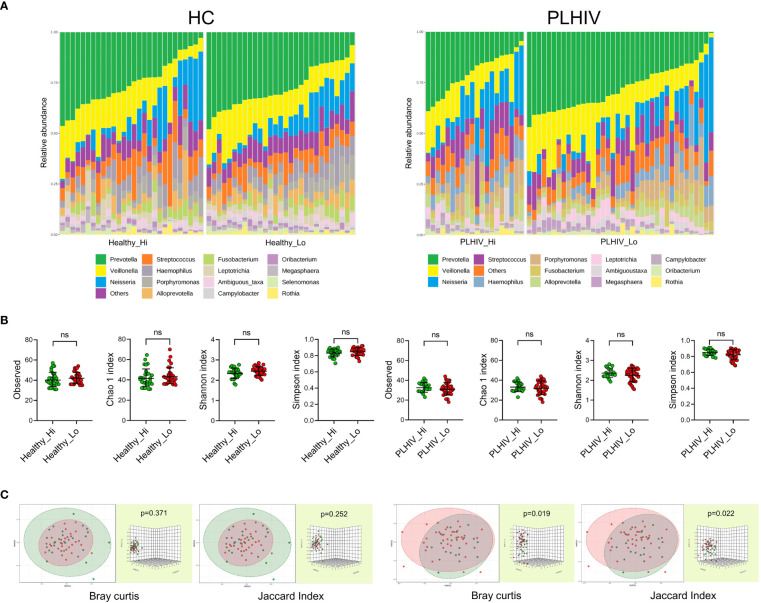
The oral microbiome richness and diversity and interpersonal variations in High- *vs*. Low responders. **(A)** Microbiome composition profiles at the genus level in low- respectively high-responders in the healthy- and PLHIV participants. It illustrates the frequently occurring genera, the “top 15” are present in all subjects in both subgroups of healthy and PLHIV, and the overall dominance of *Prevotella* (green), *Veillonella* (yellow), and *Neisseria* (blue). Vertical bars represent individual samples. **(B)** Scatterplots of alpha diversity of Observed and Chao1 indices of the ASV abundance, Shannon and Simpson’s indices of the diversity of ASV among the participants. Lines and error bars indicate geographic means and standard deviation. **(C)** Non-metric multidimensional scaling (NMDS) plots visualising the beta diversity represented with Bray-Curtis dissimilarity distances and Jaccard index, validated by PERMANOVA test. ns, not significant.

We next investigated if the oral microbiome composition correlated with the vaccine responses noted in these participants. Baseline saliva samples were sequenced to address this question by subjecting salivary DNA to Illumina 16S rRNA gene sequencing (V3-V4 region). Output data in ASV-classified format were used for all downstream analysis. As shown, the salivary microbiome composition of healthy and PLHIV at genus level showed an overall dominance of *Prevotella*, *Veillonella*, and *Neisseria*, with the top identified 15 genera present in all participants (**Figure 1A**). There were no significant differences in richness and evenness in the microbial communities between High- *vs*. Low-responders in either cohort (alpha diversity: Observed and Chao1 indices or Shannon and Simpson’s indices) (**Figure 1B**). Consistently with previously reports (15), higher microbial diversity (both alpha and beta) was found in HC as compared to the PLHIV participants (**Figure S2**). The beta diversity analysis further indicated that there were interpersonal variations between the high- and low-responders in PLHIV participants (Bray Curtis and PERMANOVA: p=0.019; Jaccard index and PERMANOVA: p=0.022) as shown by the non-metric multidimensional scaling (NMDS)-based Bray-Curtis dissimilarity distances and Jaccard index **(** **Figure 1C**, left), while no significant difference was noted between low and high responder in HC participants (Bray Curtis and PERMANOVA: p=0.371; Jaccard index PERMANOVA: p=0.252) (**Figure 1C**, right).

### Taxonomic differences of oral microbiome in Low- and High-responders

**Figure 2:**
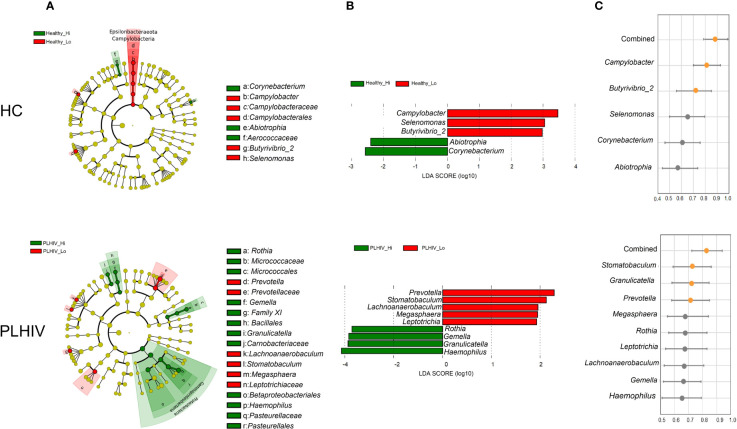
Oral microbial signatures associated with long-term antibody responses in HC and PLWH. **(A)** LEfSe’s cladogram shows the taxonomic levels, with the outer circle representing the phyla and the inner circle the genera. Each circle represents a taxa member within that taxonomic level. Green label indicates the high responders and red label the low responders. **(B)** Linear discriminant analysis (LDA) effect size analysis (LEfSe) identified the most differentially abundant genera between high and low responders in the healthy and PLHIV, respectively (P <.05; LDA score > 2). High responder-associated genera are indicated with negative LDA scores (green), and low responder-associated genera indicated with positive LDA scores (red). **(C)** The Area Under the Receiver Operating Characteristic (AUROC) scores show the predictive values of the individual microbial feature, or the combined significant features to predict the type of response e.g. Healthy_Hi (n=28) *vs*. Healthy_Lo (n=29), or PLHIV_Hi (n=20) *vs* PLHIV_Lo (n=38) among all vaccinees. Orange dots indicate AUC > 0.70 and gray dots indicate AUC < 0.70.

To find the bacteria taxa that were differentially abundant between the High- and Low responders, Linear discriminant analysis Effect Size (LEfSe) analysis was applied for the HC and PLHIV data separately. Shown in **Figure 2A**, the analysis of the taxonomic cladograms identified several significant differentially abundant taxa between the groups. The assigned LDA scores further showed that Healthy Low-responders (Healthy_Lo) (**Figure 2A**) had an increased abundance of *Campylobacter*, *Selenomonas and Butyrivibrio 2*, and reduced abundance of *Abiotrophia* and *Corynebacterium*, as compared to the High responders (Healthy_Hi). As for the PLHIV participants (**Figure 2B**), Low-responders (PLHIV_Lo) had an increased abundance of *Prevotella*, *Stomatobaculum, Lachnoanaerobaculum, Megasphaera* and *Leptotrichia*, while *Rothia, Granulicatella, Haemophilus*, and *Gemella* were lower, as compared to High-responders (PLHIV_Hi).

We further determined the predictive values of the identified microbial features as a validation and to address how well they could distinguish the outcome of vaccination responses in the participants. The results obtained from area under the receiver operating characteristic curves (AUROC) indicated that these microbial features individually (*Campylobacter* and *Butyrivibrio 2*) provided predictive value of 0.810 and 0.703 respectively (AUROC; 95% CI, p<0.0001 and p<0.005) for salivary spike-IgG responses among HC participants. When combining all five significant bacteria features, the AUROC score increased to 0.879 (p<0.0001) (**Figure 2**, right panel). Consistent with these findings, we found in PLHIV that *Stamotabaculum*, *Granulicatella, Prevotella* individually yielded predictive values of 0.710, 0705 to 0.703, respectively (p=0.008, 0.0112, 0.0107, respectively). When combining all nine significant bacteria from PLHIV, the AUC score increased to 0.82 (p<0.05) (**Figure 2B**, right panel).

**Figure 3:**
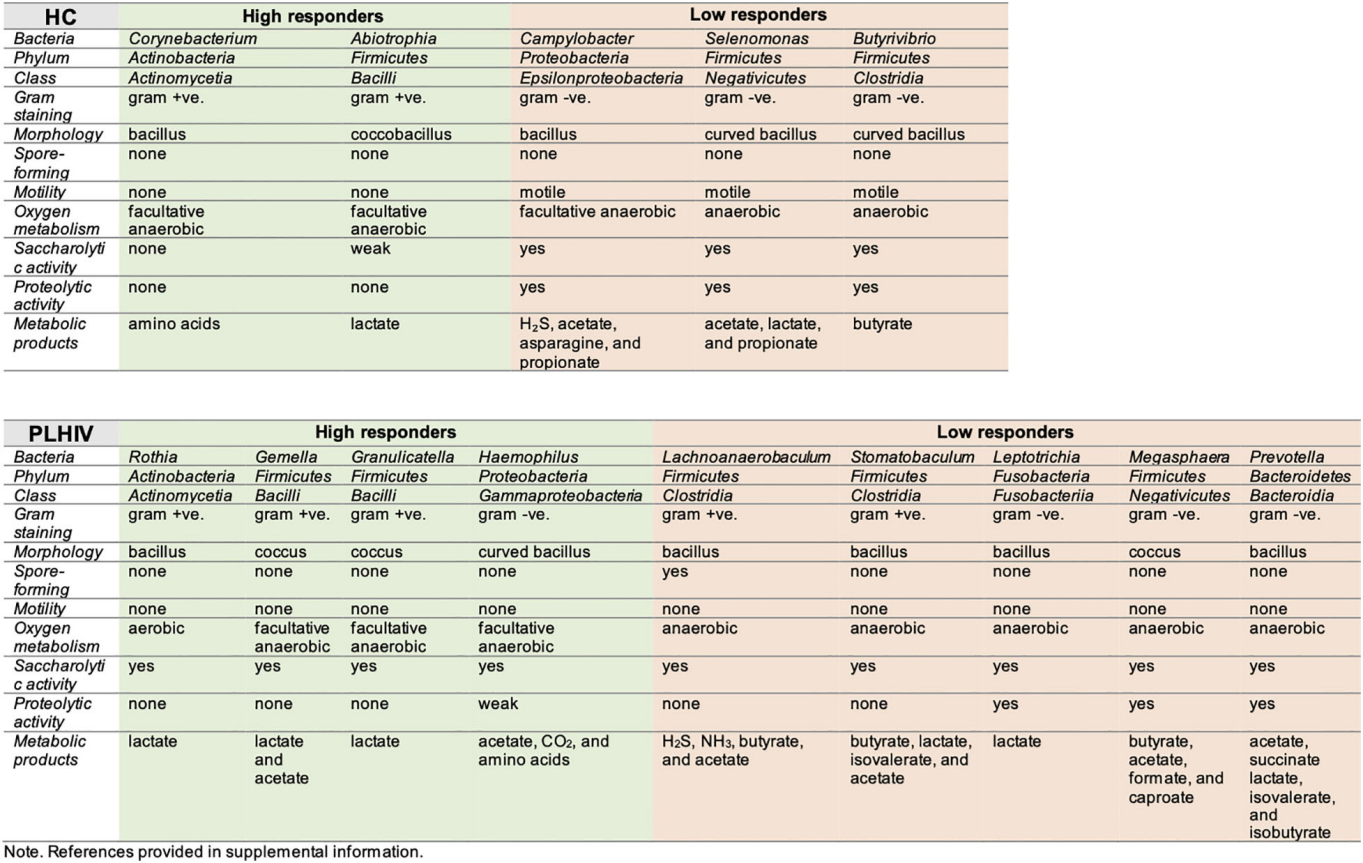
Overview of microbial features, enriched in high- and low-responders to COVID-19 vaccine, per study group (HC and PLHIV).

**Figure 4:**
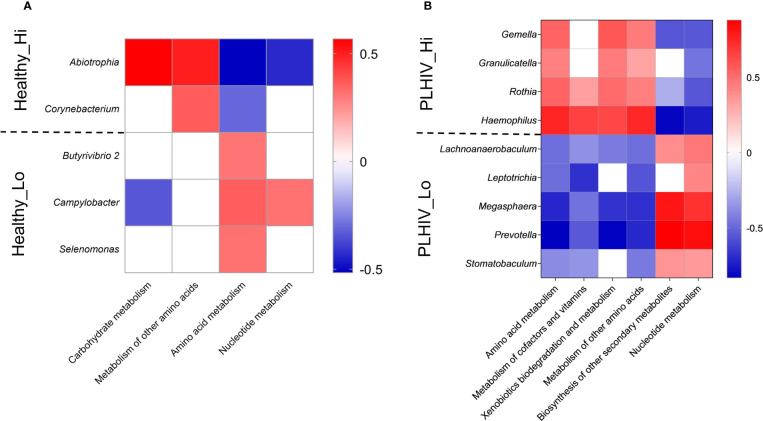
Oral microbial signature and KEGG metabolism associated with High *vs*. Low responders in HC **(A)** or PLHIV **(B)**. Heatmap representation of KEGG metabolism pathways result on the microbial signatures of High (Hi) and Low responder (Low) that were selected by LEfSe v1.1.01 (linear discriminant analysis (LDA) at genus level (score > 2, P < 0.05) identified in Healthy and PLHIV participants. Red = positive correlations and blue = negative correlations by Spearman correlation (rho) at significance level of p < 0.05 and FRD <0.05.

We considered that there could be functional resemblances beyond the identified bacteria taxa. Striking, we observed functional associations indicating that, among Low-responders of HC as well as of PLHIV, the enriched taxa were mainly of anaerobic, gram-negative (lipopolysaccharide LPS+) bacteria species with known proteolytic activities (**Figure 3**). In a subsequent KEGG-pathway analysis (**Figure 4** **)**, they also showed significant positive associations with processes of amino acid metabolism, nucleotide metabolism and biosynthesis of other secondary metabolism (p < 0.05, FDR <0.05). On the contrary, in High-responders of both HC and PLHIV, the enriched species were instead mainly gram-positive bacteria of facultative genera with rather limited proteolytic activities. These bacteria were positively associated with carbohydrate metabolism, metabolism of other amino acids, vitamin and cofactor metabolism, and xenobiotics biodegradation and metabolism (p < 0.05, FDR <0.05) (**Figure 4**). Altogether, these data suggest that oral microbiome signatures in Low-responders among both HC and PLHIV cohorts resemble those described for a dysbiotic salivary community (9), and are distinguishable from the High-responders.

## Discussion

The microbiota is fundamental for health and the evolution of the immune system with the microbiota is interconnected (13). We here describe the baseline oral microbiome composition with immunogenicity follow-up in a longitudinal cohort of healthy or people living with HIV vaccinated with BNT162b2 mRNA vaccine. We found that numerous features in the oral microbiome, including the diversity, specific bacteria taxa, and functions, correlated significantly with the immunogenicity of mRNA COVID-19 vaccination - in a primary anatomical site that needs immune protection against the SARS-CoV-2 infection (1). Our finding is in line with recent studies indicating that the gut microbiome or antibiotics usage could influence the systemic immunogenicity of the same mRNA vaccine (11) studied in the present study. We found that the durability of the salivary as well as the serum immunity through the mRNA-vaccination (two doses) lasted for at least six months. But it was negatively affected by the presence of a dysbiotic oral microbiome signature in both healthy and HIV participants who participated in our clinical trial (16). Here, we identified that the oral microbiota signature of high responders in HC and PLHIV individuals harboured significantly higher abundances of gram-positive facultative anaerobes saccharolytic bacteria, particularly *Corynebacterium* spp., *Abiotrophia* spp., *Rothia* spp., *Gemella* spp., and *Granulicatella* spp. In contrast, the low responders showed significantly higher abundances of gram-negative rod-shaped anaerobic proteolytic bacteria, including *Campylobacter* spp., *Selenomonas* spp., *Butyrivibrio* spp., *Leptotrichia* spp., *Megasphaera* spp., and *Prevotella* spp. Our results further indicate that a combined oral bacterial panel has the highest ability to predict the antibody magnitude and duration in saliva following the mRNA vaccination, which is consistent with the recent gut microbiome study on a one-month follow-up of COVID-19 vaccinees (11). Besides that, our findings also suggest that the durability of vaccine-induced immunity in the oral cavity could be influenced by the baseline oral microbiome of the vaccinees up to six months.

That the oral microbiome community might regulate local vaccine-induced mucosal immunity is intriguing and to our knowledge, similar results have not been reported before for other COVID-vaccines. The findings are of interest for vaccine strategies that aim at improving the mucosal immune memory. Although the precise mechanistic role of oral microbiota in vaccine response is unknown, several potential mechanisms could explain the link between deferentially enriched oral taxa and the persistence of salivary spike-IgG in the vaccinees. While most salivary IgG antibodies are derived from the bloodstream by passive leakage of the periodontal epithelium, there can also be some local production by salivary gland plasma cells (17). Data from molecular studies suggest that extracellular receptors such as toll-like receptor 2 (TLR2) and cytoplasmic receptors such as nucleotide-binding oligomerization domain protein 2 (NOD2) can recognize Gram-positive peptide fragments or metabolites released by bacteria that trigger the NFκB pathway (18). This pathway induces further phosphorylation of tight junction proteins, which promotes the expression of polymeric Ig receptor (pIgR), controlling the rate of production, translocation, and secretion of salivary immunoglobulins. Therefore, the high abundance of Gram-positive facultative bacteria in high responders may explain the durability of salivary spike IgG levels. Previous clinical studies have also shown an inverse relationship between oral diseases such as tooth decay, oral mucositis, and salivary immunoglobulin antibodies (19, 20). Further, pathobionts “formerly known as periodontitis-associated bacteria” and abundant also in patients with edentulism, play unique and synergistic roles in dysbiosis of oral microbiota (21). Intriguingly, such pathobionts, especially the strictly anaerobe LPS-producing rods (*Campylobacter* spp., *Selenomonas* spp., *Butyrivibrio* spp., *Leptotrichia* spp., *Megasphaera* spp., and *Prevotella* spp.) are enriched in the saliva of the low responders. These pathobionts are known for potent proteolytic activity that can break down immunoglobulins, complements, and other innate defence proteins (22), and an inflammation-type dysbiosis has similarly been associates with long COVID (23). Whether oral health-related interventions or inhibition of specific microbial adhesion, pro-inflammatory mechanisms can further improve the duration of protective antibodies in the oral cavity during systemic or even mucosal vaccination, therefore deserves further investigation.

Our study design had considered age, sex, and BMI, which are all associated with vaccine efficacy against SARS-CoV-2 (24), as well as antibiotic use that could reduce COVID-19 vaccine immunogenicity (12). Those with exposure to SARS-CoV-2 infection before and during the study were omitted to reduce the potential bias of natural infection affecting vaccine-related antibody levels. All test platforms and clinical trial related procedures were also highly standardised and monitored (4, 14, 16). The present study is not without limitations, only baseline microbiome timepoint was characterised only, the sample size is relatively small, oral health data are lacking, the microbiome analysis was a targeted approach, and only binding IgG antibodies up to six-months after vaccination were measured.

In conclusion, our study is the first to demonstrate that oral microbiome may have a role in maintaining the long-term antibody persistence in saliva and in blood after COVID-19 mRNA vaccination. Including oral health- or microbiome-targeted interventions to improve the long-term mucosal memory after vaccination should be further explored.

## Materials and methods

### Study participants

Among 177 participants, including healthy controls (HC) or people living with HIV (PLHIV) who received two doses of mRNA vaccine Comirnaty^®^ in COVAXID Clinical Trial (NCT04780659), 115 participants (HC, n=57; PLHIV, n=58) met study criteria for the current microbiome study. They were tested on day 0 and were confirmed negative on nasopharynx SARS-CoV-2 by PCR, and were seronegative following serological analysis with the Elecsys^®^ anti-SARS CoV 2 S assay. None had recent or ongoing antibiotic treatment. All participants received two vaccine doses on study day 1 and 21, completed all baseline samplings, and tested negative for SARS-CoV-2 nucleocapsid antibodies at 6 months. Approval has been obtained by the Swedish Medical Product Agency (ID 5.1-2021-5881) for conducting the COVAXID clinical trial, and ethical permit was granted by the Swedish Ethical Review Authority (ID 2021-00451 and 2020-06381). All participants provided written informed consents.

### Sample collection

#### Sample collection and SARS-CoV-2 antibody detection in saliva

All saliva samples were processed by a standardized protocol in the same laboratory. Briefly, unstimulated whole saliva was self-collected by fasted study participants as described earlier using standardized picture instructions (4). After five minutes of passive drooling, the saliva was aliquoted in tubes using sterile transfer pipettes. All samples were immediately placed at 4°C upon the same day and stored at -80°C. Prior to antibody analysis, saliva samples were thawed at 4°C and centrifuged at 400 xg for one min at 4°C to separate any debris. Antibody analysis was performed using inactivated saliva (56°C for 30 min) as described earlier (14). Briefly, antibodies binding to the full-length spike glycoprotein in trimeric form (S-f) as well as the S1 subunit were measured by means of a multiplex bead-based assay in the 384-well plate format. The antigens were immobilized on the surface of uniquely color-coded bead identities (IDs) (MagPlex-C, Luminex corp.), and the IDs pooled to generate the bead-array. Saliva samples were diluted 1:5 in assay buffer and incubated with the array. After cross-linking the antibody-antigen complexes, a R-phycoerythrine-conjugated anti-human IgG antibody (H10104, Invitrogen) was applied for detection of IgG bound to spike. The assay readout was performed using a FlexMap3D instrument and the Luminex xPONENT software (Luminex Corp.). Each assay run included the same set of 12 negative and 4 positive saliva controls. Positive controls were samples from convalescent individuals with mild COVID-19 showing clear reactivity to spike. Negative controls were pre- pandemic saliva samples that were used to calculate the assay specific cutoffs and inter-assay variability. The inter-assay variability, evaluated as the % CV of the 16 control samples included in each assay run, was 10.8% for Spike-f and 12% for Spike S1 on average.

#### Sample collection and SARS-CoV-2 antibody detection in serum

Serum samples were analyzed for detection of antibodies to SARS-CoV-2 spike using the quantitative Elecsys^®^ Anti-SARS-CoV-2 S test (Roche Diagnostics) (25) on the Cobas 8000 e801pro. The measuring range was between 0.40 to 250 U/mL, and the cut-off value for positive results is ≥ 0.80 U/mL Positive samples with antibody titers of >250 U/mL were re-tested following 1/10 dilution, and in some cases 1/100 dilution with the upper level of measuring range 25,000 U/mL.

## Antibody quantification and data analysis

The salivary antibody data were acquired as median fluorescence intensities (MFI) for each sample and antigen. The antigen and assay specific cutoff for positivity was calculated as the mean plus 6x standard deviation (SD) of the intensity signals of the 12 selected negative controls. The inter-assay variability was estimated for Spike-f and S1 as the average percent CV of the 16 control samples included in all 6 assay runs required to test the samples included in the current study. Statistical analyses (except microbiome analysis) were performed with Prism software v.9 (GraphPad) and SPSS version 24.0 (IBM Corp, Armonk, NY, USA) Statistics. Datasets initially underwent a data normality distribution test. Differences between groups of samples were analyzed by Mann-Whitney U test for univariate analysis. Correlations were determined using Spearman rank correlation. Two-sided p values <0.05 were considered significant.

### DNA extraction and sequencing

DNA from saliva samples has been extracted using the ZymoBIOMICS™ DNA Mini Kit (Zymo Research, Irvine, California, United States) according to the manufacturer’s instructions. Elution was performed in 60 mL of RNAse-free water and resulting DNA was stored at −20°C prior to preparation for sequencing. All samples were subsequently normalized to a standard concentration of 1 ng/mL and a volume of 15 mL (for a total of 15 ng of DNA) prior to Illumina 16S sequencing (V3-V4) at KI SciLife Laboratory on MiSeq. The 16S rRNA analysis was performed using the nf-core/ ampliseq analysis pipeline (26, 27).

#### Bioinformatics and statistics analysis

Raw RNA-Seq data were quality checked using FastQC v0.11.8 and then pre-processed using Cutadapt v2.8 to remove adapter sequences and poor-quality bases. The pre-processed sequencing reads were processed using QIIME2 v2019.10.0 and were denoised using DADA2 and converted to amplicon sequence variants (ASVs). Non-bacteria taxa e.g., eukaryota, chloroplast and mitochondria in the sequence output by DADA2 were removed. Taxonomic assignments were made using the Silva v132 annotation. DADA2 detected 2456 ASVs of which 278 bacterial taxa were recovered. A total of 2653288 feature counts were detected from all samples with an average of 23072 count per sample (range: 2101 - 318474 counts). A filter was applied to remove counts < 2 and minimum prevalence of 10% per sample with low variance (5%) (28). The total sum of scales (TSS) normalisation and subsequent rarefication for sample depth normalization were applied to create the final ASV-based feature count table for downstream analysis. Tax4Fun R package (29) was used for predicting the functional profiles. Comparative analysis of the abundance at the individual level of subgroups was done using TSS normalized, ASV-based feature count table and visualized by the miaViz 4.2 R package. Alpha diversity metrics such as Observed, Chao1, Shannon and Simpson were analysed in QIIME2 v2019.10.0. Differential microbial communities (in - between or beta diversity) were assessed by Microbiome Analyst, using Bray–Curtis- and Jaccard index-based non-metric multidimensional scaling (NMDS) and significance test of PERMANOVA. Differentially abundant genera analysis was done using Linear Discriminant Analysis Effect Size (LEfSe v1.1.01) in in the Galaxy web application (http://huttenhower.sph.harvard.edu/galaxy) (30). Correlation analysis was performed with Spearman correlation test and the receiver operating characteristic curve (ROC) was applied. The CombiROC (https://ingmbioinfo.github.io/combiroc/) was used for selection of combination of biomarker features and ROC was visualized in MATLAB. Subgroup analysis of KEGG (Kyoto Encyclopedia of Genes and Genomes) metabolisms was performed with LEfSe v1.1.01 (linear discriminant analysis (LDA) selected features at genus level (score > 2, P < 0.05), followed by Spearman correlation test (P < 0.05, FDR < 0.05).

## Data availability statement

The datasets presented in this study can be found in online repositories. The names of the repository/repositories and accession number(s) can be found below: https://www.ncbi.nlm.nih.gov/bioproject/?term=PRJNA900274.

## Ethics statement

The studies involving human participants were reviewed and approved by Swedish Ethical Review Authority. The patients/participants provided their written informed consent to participate in this study.

## Author contributions

Conception, planning, funds acquisition: PN, H-GL, PL. SA, MSC. Planning and organised clinical work: PB, PL, PC, PN, SA. Performed laboratory analyses or statistics: MG, KA-M, SN, KH, GG, MS, SR, MA, SM, GB, EP. Conclusion and interpretation of result: MG, KA-M, EP, PN, SA, MSC. Wrote first draft: MG, KA-M, MSC. Reviewed and edited final version: All co-authors. All authors contributed to the article and approved the submitted version.
