# Supplementary material for: Persistence of salivary antibody responses after COVID-19 vaccination is associated with oral microbiome variation in both healthy and people living with HIV
Source: Front Immunol. 2023 Jan 10;13:1079995. doi: 10.3389/fimmu.2022.1079995 (PMC9871925; doi:10.3389/fimmu.2022.1079995)
Supplement: Supplementary file 1 [file DataSheet_1.pdf]

Supplementary Fig 1.

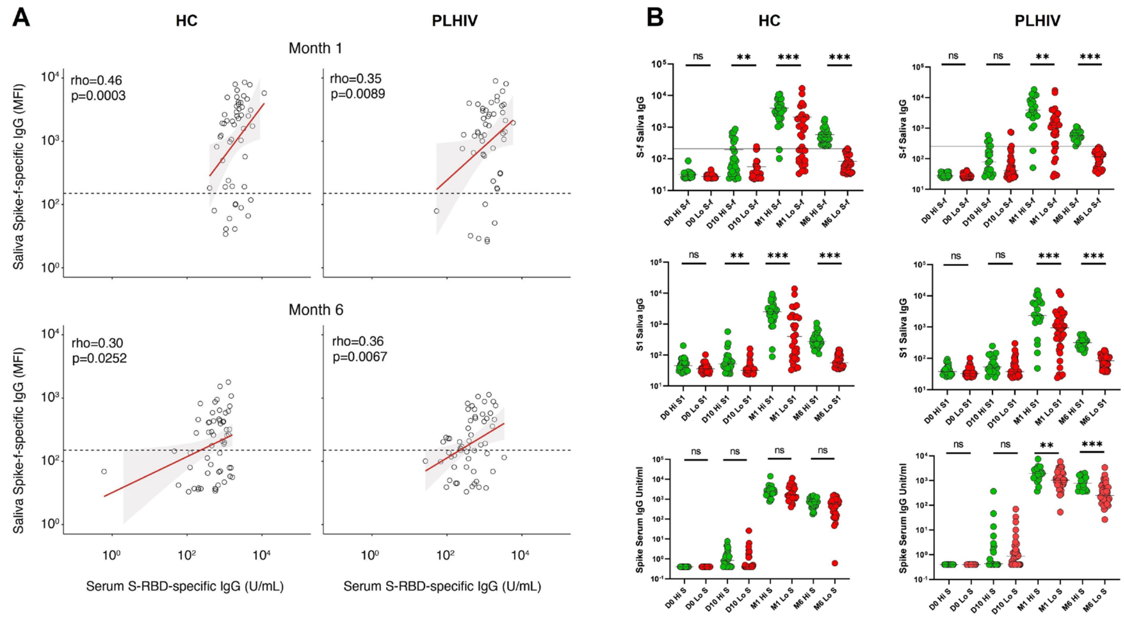

Figure S1. Development and duration of vaccine-induced spike-IgG in saliva and serum in high- vs low responders. (**A**) Correlation between the salivary and serum spike IgG among healthy participants (HC) or people living with HIV (PLHIV) on month 1 (day 35) and month 6, respectively for HC. (**B**) Spike IgG levels at day 0, day 10, day 21, and month 1 to month 6 in saliva (upper 2 panel: S-f = trimeric spike antigen, S-1 = S-1 spike antigen) and in serum (lower 1 panel: spike antigen). The Mann-Whitney U test was used to test significance. *** p<0.001, ** p<0.01. ns = not significant.

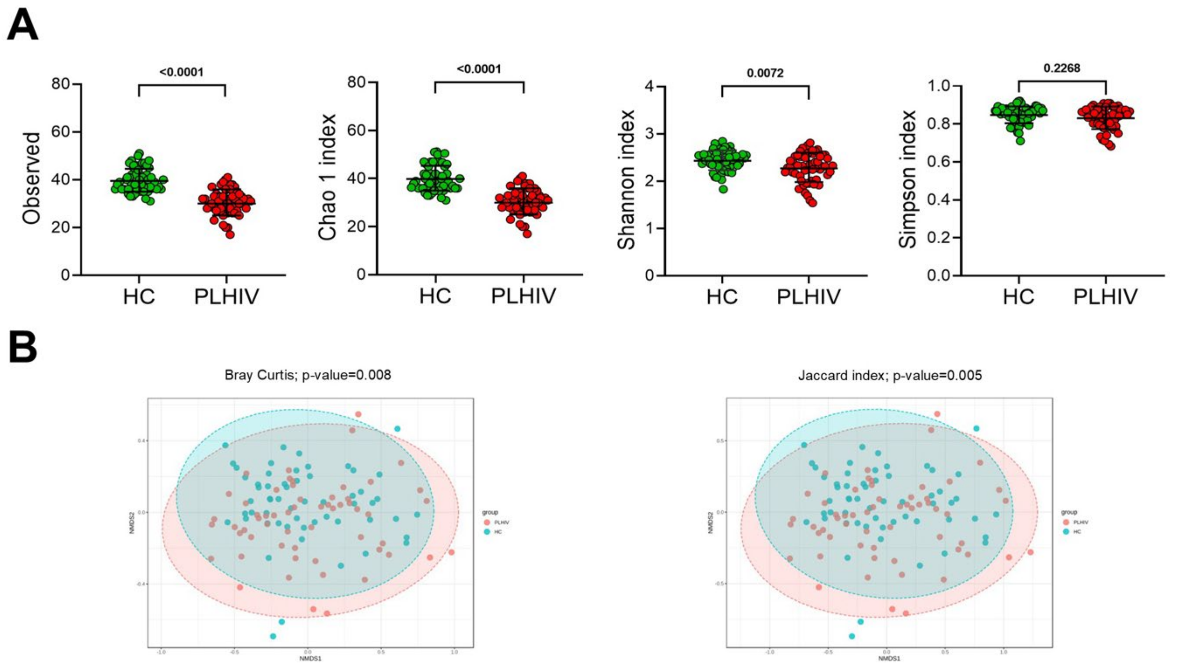

Figure S2: Alpha and beta diversity of bacterial genera between participants (HC) or people living with HIV (PLHIV). **(A)** Alpha diversity was assessed by observed, Chao1, Shannon and Simpson matrices, followed by Mann-Whitney U test **(B)** Beta diversity was assessed by Bray-Curtis dissimilarity distances and Jaccard index, validated by PERMANOVA test and

visualized by non-metric multidimensional scaling (NMDS). p<0.05 indicates statistical significance. ns = not significant.

Supporting references to Table 3

1. Kwaszewska A, Sobiś-Glinkowska M, Szewczyk EM: **Cohabitation--relationships of corynebacteria and staphylococci on human skin**. *Folia Microbiol (Praha)* 2014, **59**(6):495-502.
2. Ezaki T, Kawamura Y: **Abiotrophia**. In: *Bergey's Manual of Systematics of Archaea and Bacteria.* edn.: 1-4.
3. Yeow M, Liu F, Ma R, Williams TJ, Riordan SM, Zhang L: **Analyses of energy metabolism and stress defence provide insights into Campylobacter concisus growth and pathogenicity**. *Gut Pathog* 2020, **12**:13.
4. Marchandin H, Teyssier C, Campos J, Jean-Pierre H, Roger F, Gay B, Carlier JP, Jumas-Bilak E: **Negativicoccus succinicivorans gen. nov., sp. nov., isolated from human clinical samples, emended description of the family Veillonellaceae and description of Negativicutes classis nov., Selenomonadales ord. nov. and Acidaminococcaceae fam. nov. in the bacterial phylum Firmicutes**. *Int J Syst Evol Microbiol* 2010, **60**(Pt 6):1271-1279.
5. Moon CD, Pacheco DM, Kelly WJ, Leahy SC, Li D, Kopecny J, Attwood GT: **Reclassification of Clostridium proteoclasticum as Butyrivibrio proteoclasticus comb. nov., a butyrate-producing ruminal bacterium**. *Int J Syst Evol Microbiol* 2008, **58**(Pt 9):2041-2045.
6. GEORG LK, BROWN JM: **Rothia, gen. nov. an aerobic genus of the family Actinomycetaceae**. *International Journal of Systematic and Evolutionary Microbiology* 1967, **17**(1):79-88.
7. Aguirre M, Morrison D, Cookson BD, Gay FW, Collins MD: **Phenotypic and phylogenetic characterization of some Gemella-like organisms from human infections: description of Dolosigranulum pigrum gen. nov., sp. nov**. *J Appl Bacteriol* 1993, **75**(6):608-612.
8. Christensen JJ, Facklam RR: **Granulicatella and Abiotrophia species from human clinical specimens**. *J Clin Microbiol* 2001, **39**(10):3520-3523.
9. Tchatchouang S, Nzouankeu A, Hong E, Terrade A, Denizon M, Deghmane AE, Ndiang SMT, Pefura-Yone EW, Penlap Beng V, Njouom R *et al*: **Analysis of Haemophilus species in patients with respiratory tract infections in Yaoundé, Cameroon**. *Int J Infect Dis* 2020, **100**:12-20.
10. Hedberg ME, Moore ERB, Svensson-Stadler L, Hörstedt P, Baranov V, Hernell O, Wai SN, Hammarström S, Hammarström ML: **Lachnoanaerobaculum gen. nov., a new genus in the Lachnospiraceae: characterization of Lachnoanaerobaculum umeaense gen. nov., sp. nov., isolated from the human small intestine, and Lachnoanaerobaculum orale sp. nov., isolated from saliva, and reclassification of Eubacterium saburreum (Prevot 1966) Holdeman and Moore 1970 as Lachnoanaerobaculum saburreum comb. nov**. *Int J Syst Evol Microbiol* 2012, **62**(Pt 11):2685-2690.
11. Sizova MV, Muller P, Panikov N, Mandalakis M, Hohmann T, Hazen A, Fowle W, Prozorov T, Bazylinski DA, Epstein SS: **Stomatobaculum longum gen. nov., sp. nov., an obligately anaerobic bacterium from the human oral cavity**. *Int J Syst Evol Microbiol* 2013, **63**(Pt 4):1450-1456.
12. Eribe ERK, Paster BJ, Caugant DA, Dewhirst FE, Stromberg VK, Lacy GH, Olsen I: **Genetic diversity of Leptotrichia and description of Leptotrichia goodfellowii sp. nov., Leptotrichia hofstadii sp. nov., Leptotrichia shahii sp. nov. and Leptotrichia wadei sp. nov**. *Int J Syst Evol Microbiol* 2004, **54**(Pt 2):583-592.
13. Shetty SA, Marathe NP, Lanjekar V, Ranade D, Shouche YS: **Comparative genome analysis of Megasphaera sp. reveals niche specialization and its potential role in the human gut**. *PLoS One* 2013, **8**(11):e79353.

14. Downes J, Wade WG: **Prevotella fusca sp. nov. and Prevotella scopos sp. nov., isolated from the human oral cavity**. *Int J Syst Evol Microbiol* 2011, **61**(Pt 4):854-858.
